# Craniocaudal cyclic load improves risk assessment of lumbar pedicle screw loosening: finite element analysis based on computer tomography

**DOI:** 10.3389/fbioe.2025.1542352

**Published:** 2025-03-24

**Authors:** Chenyu Jiang, Hanqiang Ouyang, Yali Li, Ning Lang, Yan Zhang, Liang Jiang, Huishu Yuan

**Affiliations:** ^1^ Department of Radiology, Peking University Third Hospital, Beijing, China; ^2^ Department of Orthopaedics, Peking University Third Hospital, Beijing, China; ^3^ Engineering Research Center of Bone and Joint Precision Medicine, Beijing, China

**Keywords:** osteoporosis, biomechanical analysis, finite element analysis, pedicle, screw loosening

## Abstract

**Background:**

Pedicle screw loosening (PSL) is a frequent complication in osteoporotic patients undergoing spinal fixation, yet effective risk assessment methods are limited. This study explores the impact of craniocaudal cyclic load on pedicle screw fixation strength using computed tomography-based finite element analysis (CT-FEA) and evaluates its predictive value for PSL.

**Methods:**

A total of 23 PSL cases (7 men and 16 women) and 29 matched controls were analyzed using CT-FEA. Both a simple axial pullout load and a pullout load with a preset craniocaudal cyclic load were applied to calculate the pullout force. Hounsfield unit (HU) values and volumetric bone mineral density (vBMD) of the screw trajectory were also assessed for osteoporosis evaluation. The pullout force and osteoporotic assessment value were compared between PSL and controls.

**Results:**

Craniocaudal cyclic loading significantly reduced the pullout force (924.3 ± 195.1 N vs. 745.2 ± 188.7 N, p < 0.0001). The PSL group had a lower pullout force under cyclic load (629.6 ± 188.2 N vs. 836.9 ± 131.6 N, p < 0.0001) and lower HU value of screw trajectories (183.7 ± 42.6 vs. 206.7 ± 29.72, p = 0.026) than controls, while simple axial pullout force and vBMD showed no significant differences. Receiver operating characteristic (ROC) analysis indicated that pullout force under cyclic load (AUC = 0.806) was a better predictor of PSL than HU values (AUC = 0.629).

**Conclusion:**

This study demonstrates the critical role of craniocaudal cyclic loading in pedicle screw fixation strength and its predictive value for PSL. Craniocaudal cyclic load reduces screw fixation strength significantly. Pullout force under cyclic load assessed by CT-FEA enhances the predictive accuracy for PSL risk.

## Introduction

Pedicle screw fixation is widely used for the stabilization of the postoperative spine for various conditions such as degenerative disease, trauma, tumor, infection, and deformity (Bydon et al., 2015). Pedicle screw loosening (PSL) is a recurrent complication of posterior fixation surgery that has been reported in many studies at a rate ranging from <1% to 54.6% in non-osteoporotic bone (Galbusera et al., 2015) and up to 60% in patients with osteoporosis (El et al., 2013).

Osteoporosis has been considered a main cause of PSL, where a vertebra has a markedly low capability to sustain stresses without failure (Ponnusamy et al., 2011). Yuan et al. took osteoporosis as an independent risk factor of PSL (odds ratio (OR): 8.19, 95% confidence interval (CI): 2.40–27.97) (Yuan et al., 2023). However, some studies have suggested that vertebral body Hounsfield unit (HU) value or bone mineral density (BMD) are not effective predictors of PSL, but HU or BMD around the screw trajectory are (Ishikawa et al., 2018; Xu et al., 2020; Li et al., 2023; Wichmann et al., 2015; Zou et al., 2020). These studies suggest that the fixation strength of pedicle screws may be related to the degree of osteoporosis, specifically around screw trajectories.

The external load of the internal fixation device may be another critical factor of PSL. The axial pullout force was considered the main mechanical index of PSL in both traditional *in vitro* biomechanical tests and finite element analysis (FEA) (Wichmann et al., 2015; Chevalier et al., 2021; Widmer et al., 2020; Wray et al., 2015; Molinari et al., 2021; Yao et al., 2021; Sensale et al., 2021). However, recent studies have shown that the axial pullout force was not correlated with screw loosening (Fasser et al., 2022; Song et al., 2023). A previous *in vivo* study measured the primary load of daily ambulation on the screws in the craniocaudal direction by integrating load sensors into the internal spinal fixator (Graichen et al., 1996). Due to the patient’s daily activities or even breathing, the screws are subjected to cyclic external loads (Rohlmann and Graichen, 1997). Therefore, many studies have speculated that the cyclic load in the craniocaudal direction may be a potential cause of PSL (Song et al., 2023; Kueny et al., 2014; Grevenstein et al., 2020). Song F et al. used the FE method to demonstrate the effects of cyclic loading on screw loosening in an osteoporotic population (Song et al., 2023). However, the effect of such craniocaudal cyclic load has not been verified in screw trajectories where clinical PSL occurs.

Hence, the present study sought to obtain screw trajectory data through pre- and postoperative CT image registration and construct a patient-specific vertebrae-screw FE model to investigate the influence of craniocaudal cyclic load on pedicle screw fixation strength and examine its predictability in PSL.

## Methods

This study was approved by the institutional review board, and written informed consent was waived due to retrospective design. A total of 143 patients who underwent revision surgery for internal fixation due to lumbar internal fixation device disorders in our institution from January 2020 to December 2022 were initially analyzed. The subject’s inclusion criteria were as follows: 1) postoperative imaging or revision surgery records suggest the presence of PSL; 2) instrumentation from L3 to L5 in patients aged >50 years at the time of surgery; 3) minimal follow-up time of a year. Patients with no available preoperative and postoperative image; other causes of screw loosening, such as infection or trauma; history of other spinal diseases, such as spinal deformity, infection, tumor, or other metabolic bone diseases, were excluded. Finally, 23 patients with PSL at L3 were included in the analysis, and 29 patients with pedicle crew fixation at L3–L5 in the same period without pedicle screw loosening after surgery were included as the control group (Table 1).

**TABLE 1:** The demographic characteristics of all subjects.

	PSL group (n = 23)	Control group (n = 29)	p-value
Age (years)	67.0 ± 7.0	65.3 ± 8.1	0.445
Male: female	7:16	11:18	0.682
Body mass index (kg/m^2^)	26.7 ± 3.7	26.7 ± 2.6	0.115
vBMD (mg/cm^3^)	91.6 ± 17.8	100.4 ± 16.9	0.074
Cause of pedicle screw fixation (n)			
Lumbar stenosis	14	16	
Lumbar spondylolisthesis	4	5	
Lumbar scoliosis	5	8	

PSL, pedicle screw loosening; vBMD, volumetric bone mineral density.

### Patient-specific FE modeling

**FIGURE 1:**
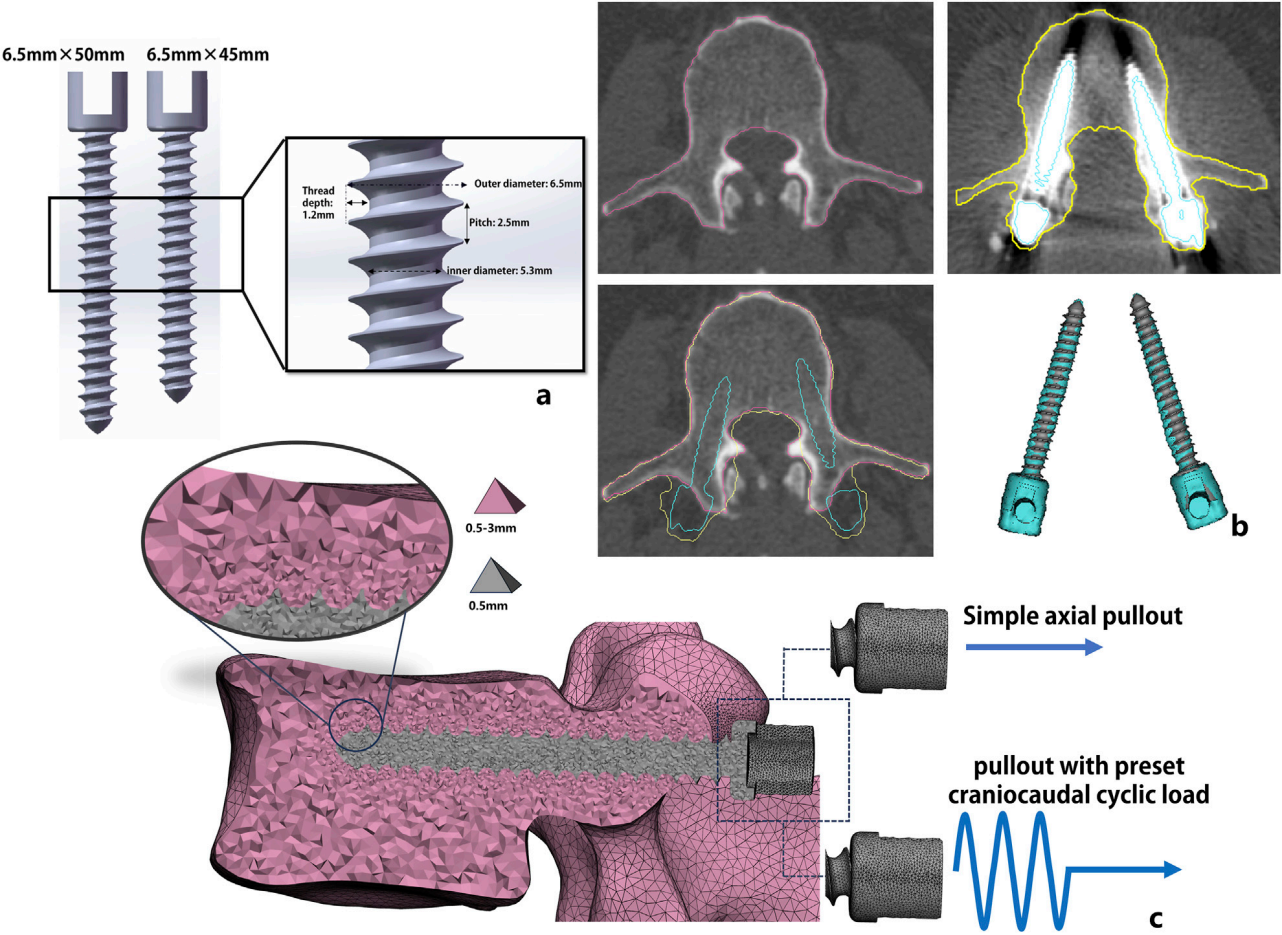
Schematic depiction of the steps involved in patient-specific model generation. Fully threaded non-cannulated pedicle screw used in the FE analysis with detailed design parameters **(a)**; Through the preoperative and postoperative CT image registration, the postoperative screw trajectory was projected into the preoperative CT image **(b)**; the patient-specific vertebra-screw model construction with tetrahedral mesh (C3D4) and the vertebral surface was fixed in all directions. Two loading conditions were applied to the screw tail: simple axial pullout displacement load and axial pullout displacement with preset craniocaudal cyclic loading **(c)**.

Pre- and postoperative CT images of all patients were imported into image postprocessing software Mimics (Materialise NV, Harislee, Belgium), where a semiautomatic segmentation was used to delineate the contours of the L3 vertebral bodies. Additionally, the screws were segmented from the postoperative images. To obtain precise patient-specific screw trajectories, we manually registered each patient’s pre- and postoperative CT images to project the real screw trajectories into the preoperative image. Due to the presence of image artifacts, the precision of the segmented screws was insufficient to directly incorporate them as three-dimensional geometric models into the finite-element model. Therefore, using CAD software, two commonly used clinical screw sizes (length 45 mm × width 6.5 mm and length 50 mm × width 6.5 mm, Figure 1A) were manually designed. The appropriate screw size was selected based on surgical records or postoperative image measurements, and the screws were registered to the preoperative vertebral bodies using a rigid iterative closest point algorithm. This process projected the actual postoperative screw trajectories onto the preoperative vertebral segmentation results (Figure 1B).

According to the method proposed by Widmer et al. (2020), the surface geometry of the vertebral body was meshed to generate a surface composed of triangular shell elements with a characteristic maximum edge length of approximately 2 mm. To accurately capture the fine details of the screw threads, the maximum edge length of the triangular mesh for the screws was set to 0.5 mm (Figure 1C). An element-size gradient adaptation algorithm was applied to transition from the fine surface mesh of the screw cavities (0.5 mm) to the coarse mesh of the vertebral surface (2 mm). The mesh distribution refinement is based on a convergence study to make it adapt to the region of interest (screw trajectory) and minimize the number of nodes to achieve a satisfactory balance between accuracy and computer resources. Finally, 250,000–400,000 4-node tetrahedral elements (C3D4) were generated, and an additional 10,000–13,000 volume elements were generated for the screw part.

### Material mapping

Young’s modulus and Poisson’s ratio of the pedicle screw (Ti-6Al-4V) were assigned as 110 Gpa and 0.33, respectively (Song et al., 2023). For a realistic representation of the patient-specific model, heterogeneous bone material properties were assigned to the created vertebral mesh. The relationship between HU and equivalent K_2_HPO_4_ densities (ρ_ash_, mg/cm^3^) was assumed to be linear and determined based on the density-calibrated phantom (Mindways Inc., Austin, TX, USA). Please refer to our previous study for details (Jiang et al., 2023). The material properties of each tetrahedral element were determined by the mean CT values or equivalent K_2_HPO_4_ densities density of the voxels within the element through an empirical material-mapping relation proposed by Morgan et al. (2003):

$$E=4730\times \rho_{app.}^{1.56}$$

A ratio between ash density and apparent density of ρ_ash_/ρ_app_ = 0.6 was assumed, and Poisson’s ratio was set to 0.3 for all elements. A linearly elastic–plastic material behavior was applied to all bone elements, where the yield stress (σ_ys_, MPa) was defined following Morgan and Keaveny (2001):

$$\sigma_{ys}=37.1\times \rho_{app}^{1.74}.$$

We adapted the plastic strain failure criterion ( $\epsilon_{\max}=0.04$ ) for the bone element to simulate the failure of the interface between bone and screw when the screw is pulled out, which demonstrated good agreement with experimental mechanical data (Widmer et al., 2020). Tetrahedrons that reached maximum plastic strain were immediately deleted to render a simplified failure behavior.

### Boundary conditions

The penalty method was set at the interface between the screw and the vertebra with a contact friction of 0.2. Rigid body properties were applied to all nodes of the screw. The inferior and superior endplates of the vertebral body were fully constrained in all directions.

Two load conditions were applied to all FE models. The first was to only apply an incremental displacement to the screw tail in the axial direction to simulate the pullout testing without considering the craniocaudal cyclic load. The other was to apply a craniocaudal cyclic load to the screw tail followed by axial pullout displacement (Figure 1C). Specifically, the amplitude curve of a sine function y = Asin (ωx + *ϕ*) was created, amplitude (A) is the craniocaudal cyclic load set at ±200 N, cyclic frequency (ω) is 2π, *ϕ* is 0, and the time span is 100 cycles. Refer to the average number of cycles used in Song F et al. study (Song et al., 2023).

All nonlinear FEA was performed with the explicit FE solver ABAQUS (ABAQUS 6.14, Simulia, Providence, RI, USA), which has the advantage of being less prone to error termination and also handles element deletion well.

### Osteoporosis evaluation

**FIGURE 2:**
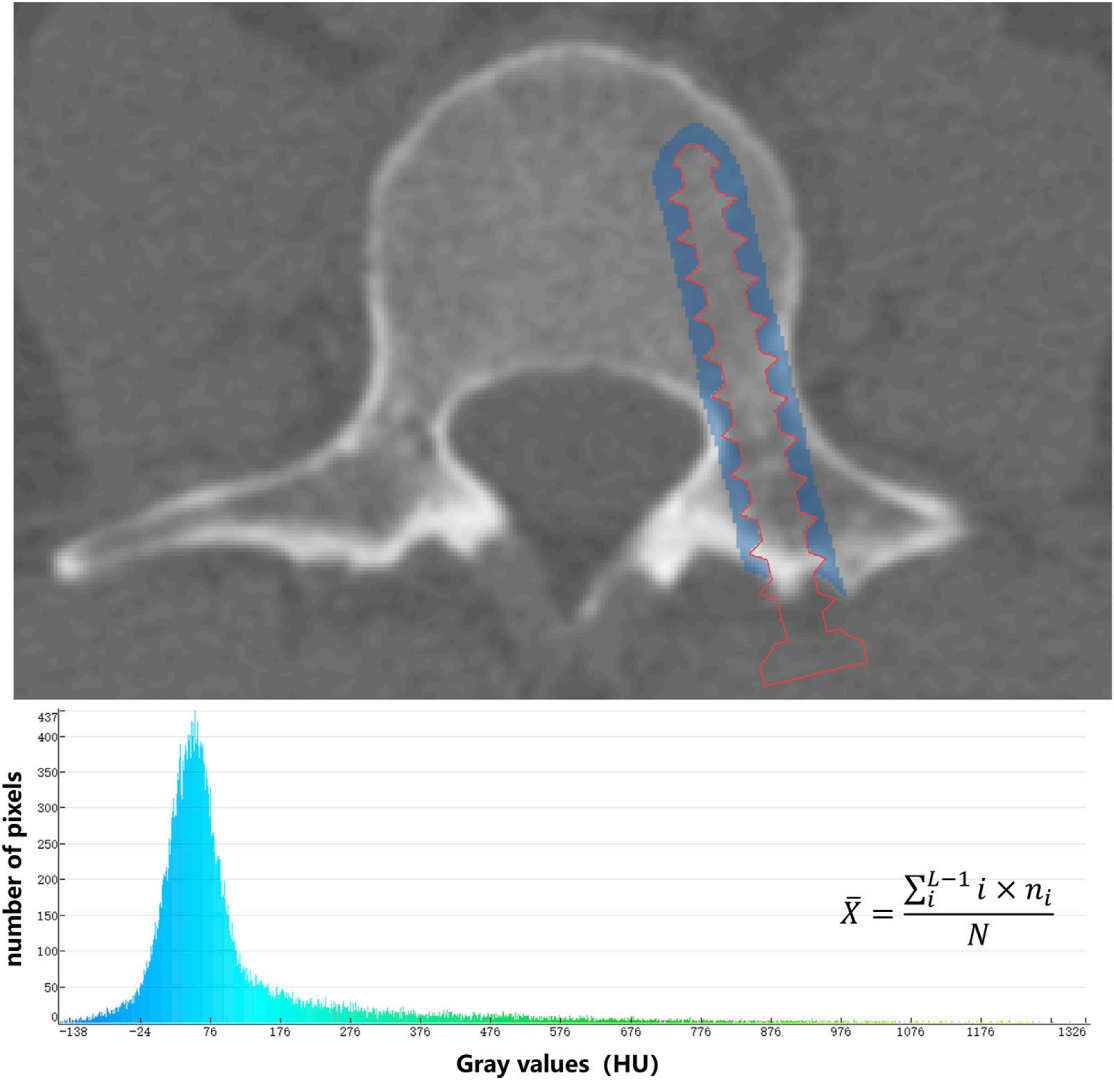
The average CT value ( $\bar{X}$ ) of the 3-mm area around the screw trajectory was measured based on the histogram. The range of gray levels of the image is [*0, L−1*]. The number of pixels with gray level *i* is *n*_*i*_, and the total number of pixels in the image is *N*.

CT images were sent to Mindways QCT Pro Version 5.0 (Mindways Sofware Inc., Austin, TX, USA) to measure the L3 trabecular vBMD (mg/cm^3^). To measure the average CT value of the screw trajectory, we used an algorithm to expand the registered screw by 3 mm. Then, through Boolean operations, we segmented an approximately cylindrical mask that is 3 mm thick around the screw trajectory. After that, we calculated the average CT value of all voxels within the mask using the gray-scale histogram (Figure 2).

### Statistical analysis

With reference to Fasser et al. (2022) and Song et al. (2023), the difference in screw fixation strength between the PSL group and the control group before and after cyclic loading was applied was approximately 68%. The test level α was set at 0.05, and the expected test efficacy 1−β was 0.80. The required sample size for each group was approximately 15 patients. A paired t-test was conducted to test for the effect of cyclic load. The HU value and pullout force were tested using the independent-samples t-test. Logistic regression was fitted to the data, and the receiver operating characteristic (ROC) curve was computed. The correlation between CT-FEA and osteoporosis assessment was compared based on Pearson correlation analysis and linear regression. The statistical analyses were performed with SPSS (SPSS 22.0, IBM Inc., Chicago, United States). The significance level was set at p < 0.05.

## Results

### Osteoporotic assessment between two groups

**FIGURE 3:**
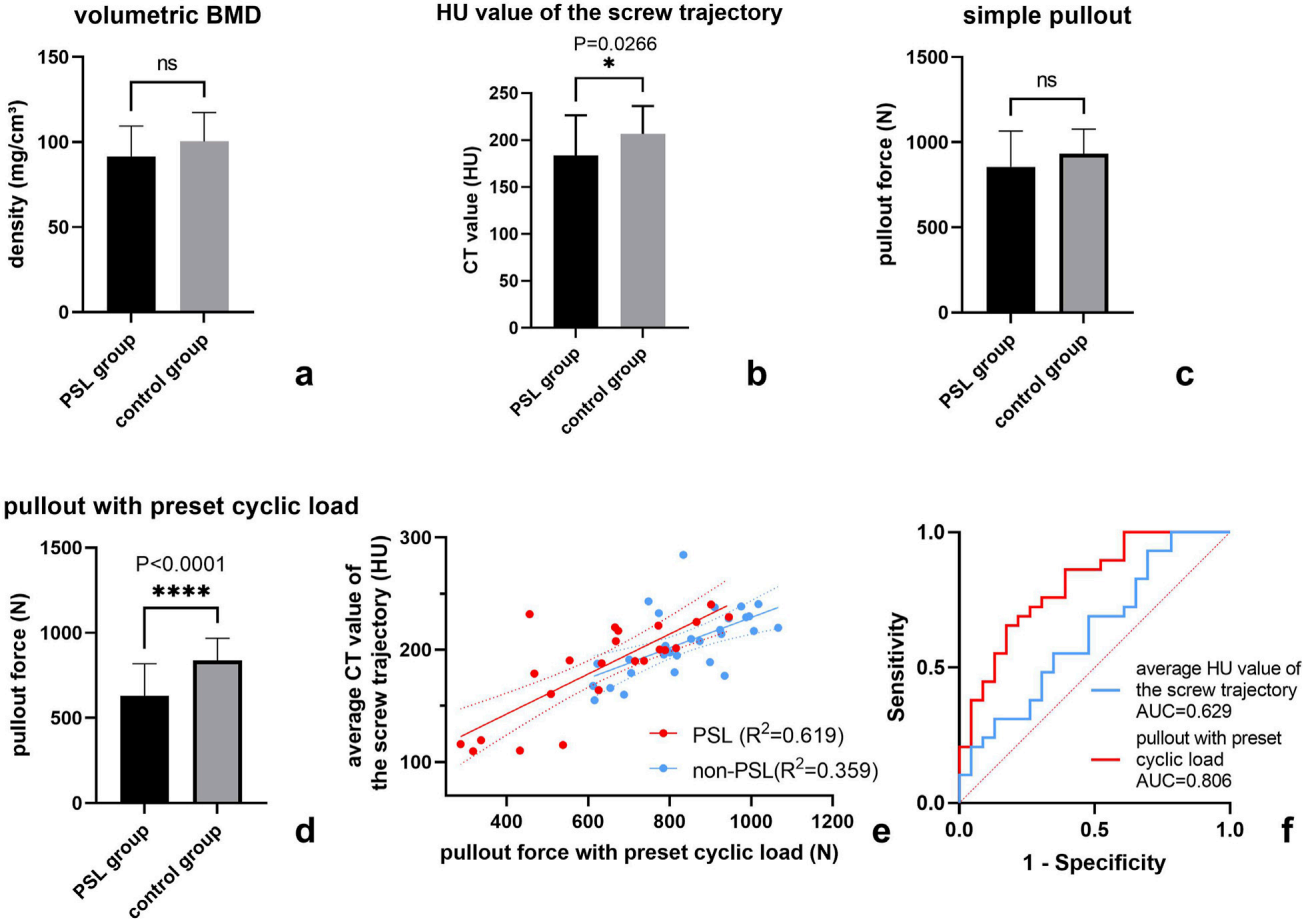
Statistical analysis of FEA-derived pullout force and osteoporotic assessment in the PSL group and the control group. The comparison of FEA-derived pullout force and osteoporotic assessment between the PSL 18 group and the control group **(a–d)**; Scatter plot of the relationship between pullout force under preset cyclic load and average CT value of screw trajectory in both PSL group and controls **(e)**; ROC analysis of FEA and HU value of screw trajectory **(f)**.

Compared to controls, the vertebral vBMD values were lower in the PSL group, although the difference was not significant (91.6 ± 17.8 vs. 100.4 ± 16.9, p = 0.074). However, the average HU values of the screw trajectories in the PSL group were significantly lower than those in the control group (183.7 ± 42.6 vs. 206.7 ± 29.72 p = 0.026, Figures 3A, B).

### Effect of cyclic load on screw pullout force

**FIGURE 4:**
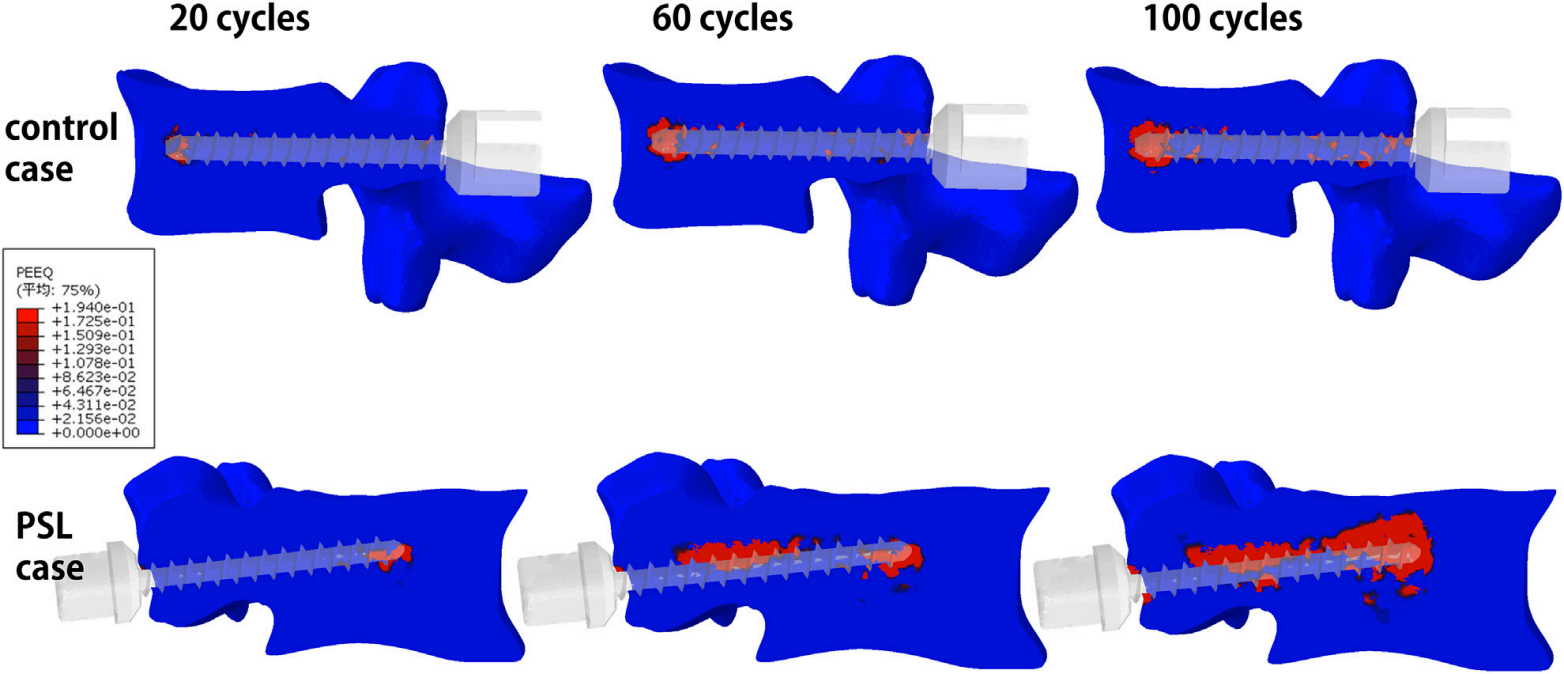
The distribution of equivalent plastic strain (PEEQ) around the screw trajectory of representative PSL case and control case after 20 cycles, 60 cycles, and 100 cycles of craniocaudal cyclic load was applied. PEEQ is mainly distributed around the tip of the screw, and more PEEQ distributed around the vertebral pedicle might be a characteristic of the PSL case.

Paired t-tests showed that 100 cycles of craniocaudal cyclic load had remarkably reduced the axial pullout force of pedicle screws (924.3 ± 195.1 N vs. 745.2 ± 188.7 N, p < 0.0001). In both the control group and the PSL group, the pullout force with preset cyclic load was significantly reduced compared with the direct simulation of screw extraction (Table 2) by 32.2% for the PSL group and 15.5% for the control group. The distribution of equivalent plastic strain (PEEQ) indicated that the yielding of bone around the screw trajectory due to cyclic loading resulted in only a minor increase around the fixed screw trajectory, while a significant progressive increase in tissue yielding was observed around the loosened screw trajectory. Furthermore, simulation results indicated that plastic strain initially concentrated around the screw tip, with increased cycles leading to gradual yielding in the pedicle region for both groups but notably more pronounced around the loosened screw trajectory (Figure 4). Moreover, there was no significant difference between the PSL and the control group in the simple axial pullout force (Figures 3C, D). However, the pullout force under cyclic load in the PSL group was significantly lower than that in the control group (629.6 ± 188.2 N vs. 836.9 ± 131.6 N, p < 0.0001, Table 2).

**TABLE 2:** The pullout force under the two loading conditions between the PSL group and the control group.

	PSL group	Control group	p-value
Simple pullout (N)	856.2 ± 209.3	933.4 ± 144.1	0.122
Pullout with preset cyclic load (N)	639.2 ± 169.4	819.4 ± 125.1	<0.0001^†^
Mean absolute difference [95% CI]	226.7 [80.84–372.5]	134.4 [23.13–245.7]	
Mean percent difference [95% CI, %]	32.20% [9.482–54.91]	15.52% [3.764–27.28]	
p-value	<0.0001^*^	<0.0001^*^	

PSL, pedicle screw loosening; CI, confidence interval; *, significant differences with paired t-test; †, significant differences with independent sample t-test.

### Correlation analysis

Based on Pearson correlation analysis, we found that the pullout force under cyclic load was significantly correlated with the average HU value of the screw trajectory instead of vertebral vBMD (Pearson r: 0.628 [0.3017–0.8232] vs. 0.234 [-0.1870–0.5825], p = 0.001 and p = 0.271). Furthermore, the correlation between pullout force under cyclic loading and the average HU value of the screw trajectory in the loosened screw trajectories was significantly higher than that in the non-loosened screw trajectories of the control group (Figure 3E).

### Prediction of PSL risk

Binary logistic regression analysis showed that the pullout force after cyclic load and the average HU value of screw trajectories were related risk factors for PSL (Table 3). In addition, we compared the predictive abilities of the two factors on PSL based on the ROC curve and found that the AUC of the pullout forces with a preset cyclic load was slightly greater than the average HU value of the screw trajectories (Table 3; Figure 3F).

**TABLE 3:** PSL risk assessment with pullout force under cyclic load and the HU value of the screw trajectory.

	OR per SD [95%CI]	AUC [95%CI]	p-value
Pullout force under cyclic load (N)	4.8 [2.1–13.6]	0.806 [0.6888–0.9244]	0.0008
Average CT value of the screw trajectory (HU)	1.9 [1.1–4.0]	0.629 [0.4763–0.7831]	0.036

PSL, pedicle screw loosening; HU, Hounsfield unit; OR, odds ratio; AUC, area under curve; 95% CI, confidence interval.

## Discussion

In this study, we proposed a novel FEA pipeline combining cyclic load in the craniocaudal direction to evaluate pedicle screw fixation strength. The results showed that the cyclic load led to a significant decrease in pedicle screw fixation strength, and the pullout forces under cyclic load were significantly different between the two groups. Compared to simple pullout force estimation by FEA and clinical osteoporotic assessment, the pullout force under cyclic load based on CT-FEA proposed in this study was helpful in improving the predictive ability of clinical PSL.

In evaluations of screw loosening risk based on FEA in previous literature, the simple axial pullout force was used as the main fixed-strength indicator (Chevalier et al., 2021; Widmer et al., 2020; Wray et al., 2015; Molinari et al., 2021; Yao et al., 2021). However, most of these studies focused on FE model validation. Although FE simulation results revealed a high correlation with *in vitro* mechanical test results (Chevalier et al., 2021; Widmer et al., 2020; Yao et al., 2021), there is still a need to validate the simple axial pullout force as an indicator of fixation strength in clinical case–control studies. A recent study by Fasser et al. (2022) using FEA indicated that axial pullout force was not a reliable predictor of clinical PSL events. Our findings corroborated this point. However, when a cyclic load was applied, the axial pullout force in the PSL group was significantly lower. This outcome is consistent with the research by Song et al. (2023), who discovered that cyclic loading was more likely to cause yielding at the screw–bone interface in osteoporotic vertebrae. These similarities suggest that the axial pullout force measured under a preset cyclic load can serve as an effective indicator in FEA for predicting screw loosening.

Although osteoporosis is a major risk factor for screw loosening (Galbusera et al., 2015), our study found no significant difference in volumetric bone mineral density (vBMD) between the PSL and control groups. This may be attributed to the method of osteoporosis assessment and a relatively small sample size. While vBMD provides a global measure of bone density, it may not fully capture the localized bone quality around the screw trajectory, which appears to be more critical for screw stability. Previous studies have also suggested that regional bone density, particularly around the screw trajectory, is a more accurate predictor of screw loosening than overall vertebral bone density (Xu et al., 2020; Li et al., 2023; Wichmann et al., 2015; Jang et al., 2019; Bredow et al., 2016). On the other hand, the significance of the results may also be limited by some natural defects of CT in osteoporosis evaluation, including susceptibility to artifacts and beam hardening effects and the need for careful calibration.

Despite this, the average HU value of the screw trajectory in the PSL group was significantly lower than that in the control group. This indicates that screw loosening is significantly related to the degree of bone mass loss around the screw trajectory. In addition, the significantly higher correlation between pullout force under cyclic loading and the average HU value of the screw trajectory in the PSL group compared to the non-loosened group suggests that bone quality around the screw trajectory plays a critical role in screw stability under cyclic loading. This finding underscores the importance of preoperative assessment of bone quality, particularly in the screw trajectory region, to identify patients at higher risk of screw loosening. Clinically, this could guide surgeons to adopt enhanced fixation techniques, such as optimizing screw placement with more resistance to cyclic load. Furthermore, these results highlight the need for future research into screw designs that better withstand cyclic loads and the development of predictive models that integrate HU values and cyclic loading simulations to improve surgical outcomes.

Previous studies have shown that the load on the pedicle screw varied from 25 N to 325 N (Rohlmann and Graichen, 1997), and Dreischarf et al. found that a unilateral screw was subjected to an average load of 250 N in the lumbar spine of a standing posture (Dreischarf et al., 2016). Hence, the cyclic load of 250 N was employed in our FE method. In terms of the number of cycles, we refer to Baluch et al. (2014) and set it at 100 cycles. Bone tissue failure caused by craniocaudal cyclic load was characterized by PEEQ, which is a selectable field with variable output results that can show the cumulative plastic strain (Song et al., 2023). Our results show that in the PSL group, the number of elements with plastic strain around the screw was significantly larger than that in the control group, and the subsequent axial pullout displacement load also resulted in a significantly reduced pullout force. Our results suggest that the plastic strain of the bone around the screw caused by cyclic loads from daily activities may be an important risk factor for screw loosening.

Several limitations of this study should be discussed here. First, our numerical models were not validated by mechanical experiments. However, our pullout FE modeling approach refers to the study of Widmer J et al. and has been validated based on cadaveric vertebrae (Widmer et al., 2020). Due to the unavailability of bone tissue specimens of the subjects and limited fresh cadavers, such *in silico* models might be very useful for understanding biomechanical behavior given various spinal conditions. Second, the properties of complex biomechanical material could not be replicated in reality by the FE model, especially when material nonlinearity was taken into consideration. Third, this study only considered the effect of the cyclic load in the craniocaudal direction on screw fixation. Under real postoperative conditions, the load subjected to internal fixation is very complicated. The cyclic load in other directions and even the rotating torque should be addressed in future screw loosening analyses and FE simulations. In addition, our sample size is relatively small. Yet, discernible results were promising, and prospective studies with larger sample sizes would reveal the broader value of FEA in clinical applications.

## Conclusion

This study demonstrates the critical role of craniocaudal cyclic loading in pedicle screw fixation strength and its predictive value for PSL. The craniocaudal cyclic load significantly reduces the screw fixation strength. Pullout force under cyclic load using CT-FEA improves clinical PSL prediction compared to simple pullout strength and osteoporotic assessment. Patient-specific FEA incorporating cyclic loading offers a promising approach to improve clinical assessment and prevention strategies for PSL.

## Data Availability

The raw data supporting the conclusions of this article will be made available by the authors, without undue reservation.
